# Chlorogenic Acid Alleviates Thiram-Induced Tibial Dyschondroplasia by Modulating Caspases, BECN1 Expression and ECM Degradation

**DOI:** 10.3390/ijms20133160

**Published:** 2019-06-28

**Authors:** Jialu Zhang, Shucheng Huang, Xiaole Tong, Lihong Zhang, Xiong Jiang, Hui Zhang, Khalid Mehmood, Jiakui Li

**Affiliations:** 1 College of Veterinary Medicine, Huazhong Agricultural University, Wuhan 430070, China; 2 University College of Veterinary & Animal Sciences, Islamia University of Bahawalpur, Bahawalpur 63100, Pakistan

**Keywords:** chlorogenic acid, growth plate, tibial dyschondroplasia, matrix mineralization, apoptosis, autophagy

## Abstract

Chlorogenic acid (CGA) is a widely applied traditional Chinese medicine ingredient which can be used for the treatment of osteoporosis. In this experiment, we investigated the potential therapeutic effect of chlorogenic acid on thiram-induced tibial dyschondroplasia (TD) and explored the underlying mechanisms that have been rarely mentioned by others yet. Performance indicator analysis and tibial parameter analysis showed that CGA exhibited a definite positive effect on thiram-induced TD chickens. In order to further explore the mechanisms underlying the positive actions of CGA, apoptotic, autophagic genes and MMPs involved in matrix mineralization of growth plate were evaluated in this study. The results showed that CGA decreased the expression of pro-apoptotic genes caspases-3 and caspases-9, leading to the reduction of apoptotic cells accumulated in growth plate. In addition, CGA also increased the level of BECN1, an important gene involved in autophagy, which benefits the survival of abnormal cells. Furthermore, CGA also increased the expression of MMP-9, MMP-10, and MMP-13, which can directly affect the ossification of bones. Altogether, these results demonstrate that CGA possesses a positive therapeutic effect on thiram-induced TD via modulating the expression of caspases and BECN1 and regulating the degradation of ECM (extracellular matrix).

## 1. Introduction

The growth plate is a region where longitudinal bone formation occurs via endochondral ossification, the principal process involved in most skeleton formation. The skeleton is generated by a transition from cartilage to bone [1]. During the development, chondrocytes in growth plate need to undergo several periods: the proliferation period, prehypertrophic period, also the called transition period, and hypertrophic period [2]. Growth plate in avian species has more cells and more thriving cell differentiation than in mammalian species [3]. After the maturation of chondrocytes, the cleavage and remodeling of extracellular matrix (ECM) are executed by matrix metalloproteinases (MMPs: which are secreted by many cells, such as epithelial cells and chondrocytes) [4], accompanied by vascular invasion, then calcification and bone formation proceed. During the normal development of growth plate, there are studies suggesting that terminally differentiated chondrocytes also need to undergo programmed cell death in order to allow normal endochondral ossification [5].

Tibial dyschondroplasia (TD) is a rapidly developing skeletal disease caused by its crowded feeding environment and rapid growth. A white, transparent, avascular, non-mineralization cartilage plug appears in the proximal growth plate, which is the most typical characteristic of TD [6]. The tibia in TD chickens is fragile and easy to break due to uncompleted mineralization. The initial symptoms are not obvious. As the disease develops, broiler chickens with TD consequently lose their dietary wishes and bilateral knee joint swelling is accompanied by hypertrophy of the humeral cortex. The initiation of the lesion is due to the failure of prehypertrophic chondrocytes to differentiate into hypertrophic chondrocytes. Moreover, there are studies that demonstrate that dysregulated apoptosis may contribute to the development of TD [7]. Caspase-3 (CASP-3) and Caspase-9 (CASP-9) are two important caspases and play critical roles in apoptosis, as executioner and initiator, respectively [8].

There are two widely accepted types of programmed cell death in cellular growth, apoptosis and autophagy. In recent years, many research articles have indicated the importance of autophagy in bone growth. It was proposed that autophagy is a critical regulator of bone growth and that FGF (Fibroblast growth factor) signaling regulates autophagy in chondrocytes [9]. Moreover, it seems like that the autophagy pathway can inhibit the apoptosis and make the terminally differentiated chondrocytes survival [10]. Beclin 1 (BECN1) is a crucial regulatory protein. It is involved in the initiation step of the autophagic process and regulates both the formation and maturation of autophagosomes. BECN1 plays a crucial role in the induction of autophagy or apoptosis (essentially, survival or death, respectively) in cells. BECN1 protein contains three different and important domains, comprising BH3 domain, coiled-coil domain (CCD) and evolutionary conserved domain (ECD), from N terminal to C terminal [11]. Pieces of evidence demonstrate that apoptosis may occur in hypertrophic chondrocytes which are adjacent to the metaphysis of long bones [12]. The apoptosis of chondrocytes might be related to extracellular matrix degradation [13,14] in which MMPs play an important role.

Chlorogenic acid (CGA) is a kind of carboxyphenolic acid formed by the condensation of quinic acid with caffeic acid in trans-cinnamic acid. It is a plant component, which is widely distributed in the plant kingdom and always can be extracted from higher dicotyledons and ferns [15]. The main sources are Eucommia, Honeysuckle, Coffee, Chrysanthemum and other plants. CGA has been reported to have many pharmacological activitie—for instance, neuroprotective effects [16,17,18,19], antioxidative effects [17,18], and renoprotective effects have been reported [20]. Recent research has also demonstrated that oral administration of chlorogenic acid can prevent osteoporosis in ovariectomized rats through the Shp2/PI3K/Akt pathway [21]. However, whether CGA protects against thiram-induced TD has not been investigated yet. In the current study, we investigated the protective effect of CGA on TD and the underlying mechanisms.

## 2. Results

### 2.1. Clinical Observation during the Animal Experiment

**Figure 1:**
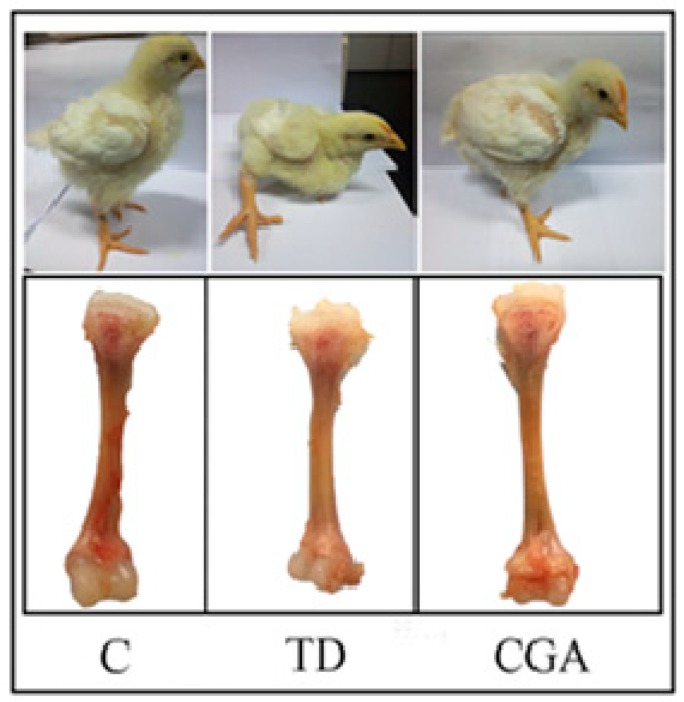
Overall clinical observation during the animal experiment. Control group represents chickens with normal diet, TD group represents chickens with normal diet plus 50 mg/kg thiram, CGA group represents the administration of chlorogenic acid after the induction of TD.

The broiler chickens in the control group had a normal diet and were able to walk freely and behave vigorously. Chickens in the TD group had a decreased appetite and apparently swollen joints of tibiae which resulted weakness, lameness, and depression. The decreasing body size is the most obvious change in the TD group. After constant chlorogenic acid treatment, the broiler’s diet was restored, the ability to walk recovered, and their body size increased to the control level (Figure 1).

### 2.2. Chlorogenic Acid Improves Performance of Chickens

**Figure 2:**
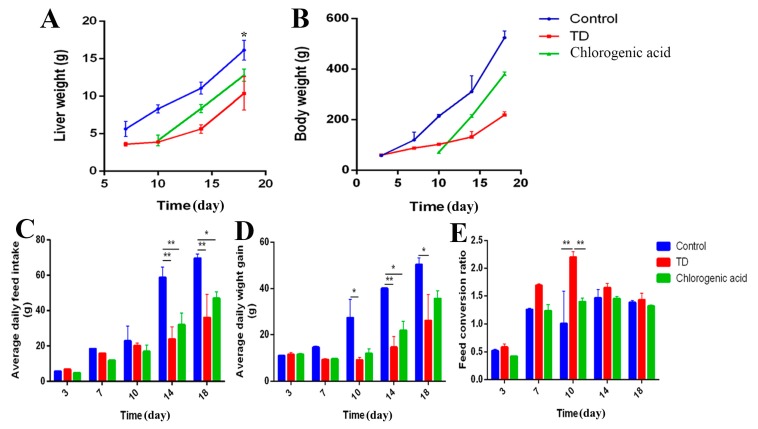
Chlorogenic acid improves performance of chickens. (**A**) liver weight measured on slaughtering days 7, 10, 14 and 18. * represents the difference between control group and TD group, * *p* < 0.05. (**B**) The body weight was recorded from the first day to the 18th, CGA can improve the body weight of TD affected chickens. (**C**–**E**) The average daily feed intake (ADFI), average daily weight gain (ADWG) and feed conversion ratio (FCR) were calculated from the first day to the 18th, TD decreased the ADFI and ADWG of chickens, the administration of CGA reverse this condition. The data are expressed as means ± SD. * *p* < 0.05, ** *p* < 0.01.

It can be found that there is a similar varying tendency among average daily feed intake, body weight and liver weight in different periods. The average feed intake, body weight, and liver weight in the TD group were lower than in the control group, which indicates a decreased growing ability. However, the feed conversion ratio in the TD group was higher than the control group and chlorogenic acid group, especially at day 10 (** *p* < 0.01), which represents a fast-growing period in TD development. After the application of chlorogenic acid, the growth of broilers was significantly promoted (*p* < 0.05), and the feed conversion ratio became normal compared with the TD group, while CGA significantly (*p* <0.05) increased the ADFI and FCR of TD-affected chickens (Figure 2). This evidence indicates that TD-affected chickens taking the chlorogenic acid display a healthy and positive growth state.

### 2.3. Chlorogenic Acid Improves Tibial Index of Chickens 

**Figure 3:**
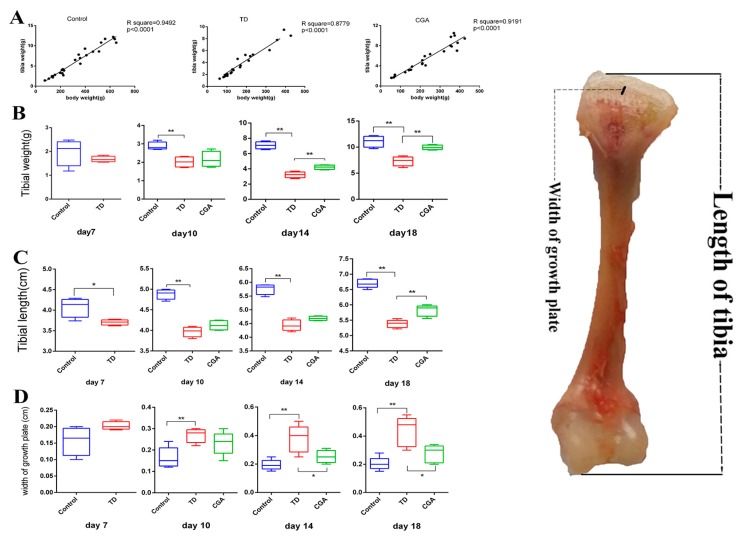
Chlorogenic acid improves tibial index of chickens. (**A**) Linear regression analysis of body weight and tibial weight among Control group, TD group and CGA group. (**B**) The tibial weight was recorded by using electronic balance at days 7, 10, 14 and 18. (**C**–**D**) The tibial length and width of growth plate were measured by using digital caliper at slaughter days 7, 10, 14 and 18. The data are expressed as means ±SD. * *p* < 0.05, ** *p* < 0.01.

Linear regression analysis showed a commonly significant (*p* < 0.05) positive correlation between body weight and tibia weight of chickens in the control, TD and CGA groups (Figure 3A). To evaluate tibial parameters precisely, tibial weight, tibial length, as well as the width of tibial growth plate were measured at days 7, 10, 14 and 18. In the TD group, tibial weight and tibial length were significantly decreased (*p* < 0.01) in comparison to the control group because of abnormal development. After the continuous treatment of CGA, tibial weight and tibial length in CGA group had significant improvement compared with the TD group (*p* < 0.01) at day 18 (tibial weight from day 14). The width of tibial growth plate has a converse tendency with tibial weight and tibial length. This index in the TD group was significantly higher than in the control group from days 10 to 18 due to delayed calcification and the appearance of avascular cartilage plug in growth plate. From day 14, CGA gradually diminished the width of tibial growth plate significantly between the TD group and CGA group (Figure 3).

### 2.4. Chlorogenic Acid Improves Antioxidant Level of Chickens

**Figure 4:**
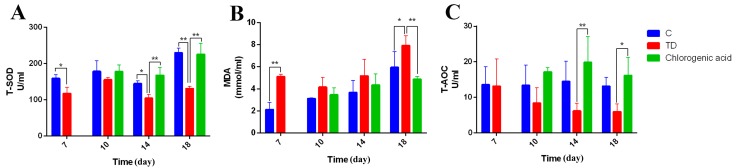
Chlorogenic acid improves antioxidant level of chickens. (**A**–**C**) Antioxidant activities analysis of serum samples in Control, TD and CGA groups on days 7, 10, 14 and 18. T-SOD represents the vitality of total superoxide dismutase, MDA represents the concentration of malonic dialdehyde, and T-AOC represents the total antioxidant capacity. The data are expressed as means ±SD. * *p* < 0.05, ** *p* < 0.01.

Antioxidant levels reflect the body’s ability to eliminate oxygen free radicals and maintain homeostasis in the body. After measuring the antioxidant indices of T-AOC, MDA and T-SOD of each group of broilers, we found that the antioxidant capacity of broilers in TD group was damaged. The levels of T-SOD and MDA in the control group and the TD group were significantly different at day 7 (* *p* < 0.05, ** *p* < 0.01). However, in the CGA group, after treatment, the activity of T-AOC and T-SOD was significantly increased compared to that of TD broilers (* *p* < 0.05, ** *p* < 0.01) from day 14, and gradually approached the level of the control group (Figure 4).

### 2.5. Observation of H&E Staining

**Figure 5:**
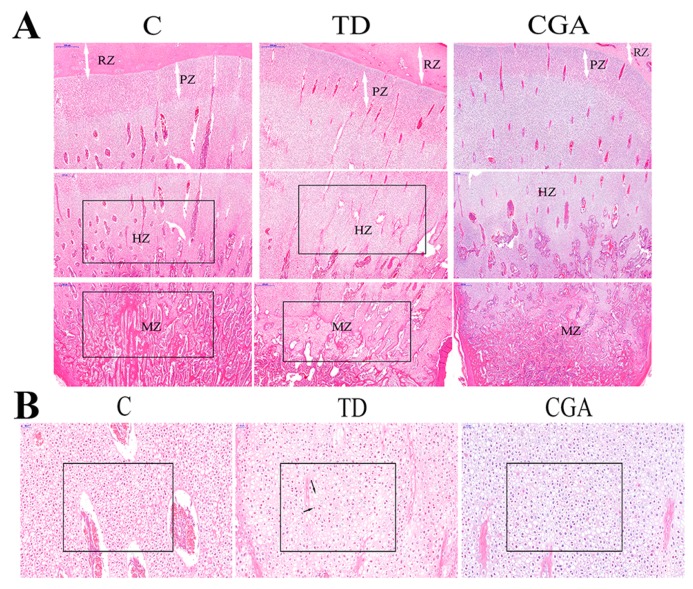
Histological examination of the tibia in broilers in three groups. C represents Control group, TD represents thiram-induced tibial dyschondroplasia group, CGA represents administration of chlorogenic acid after induction of TD. (**A**) H&E staining of tibiae containing resting zone (RZ), proliferating zone (PZ), hypertrophic zone (HZ) and mineralization zone (MZ). The scale bar is 500 μm. (**B**) HE staining of hypertrophic zone in tibiae, the long arrow indicates dead cells without nuclei and the short arrow indicates red-stained-nuclei cells in TD. The scale bar is 50 μm.

We compared the same region of tibia in three groups. The difference of cell growth can be easily found. Compared with the dense cell distribution in the control group, cells in the TD group were sparser, while there were many dead cells accumulated in the growth plate without nuclei in the TD group. Moreover, there were much more red-stained-nuclei cells in the TD group than in the control group, and the red color indicates nuclear degeneration or cellular apoptosis. After a comparison between the control group and the TD group, the TD group had a higher proportion of extracellular matrix than the control group. When it came to the calcification area, at same region of the control group and TD group, the TD group had delayed calcification compared with the control group. However, conditions became different in the CGA group where the number of cells is increased, and there are fewer red-stained cellular nuclei. Further, the proportion of extracellular matrix is decreased (Figure 5).

### 2.6. CGA Regulates Expression of Apoptotic and Autophagic Genes in Growth Plate

**Figure 6:**
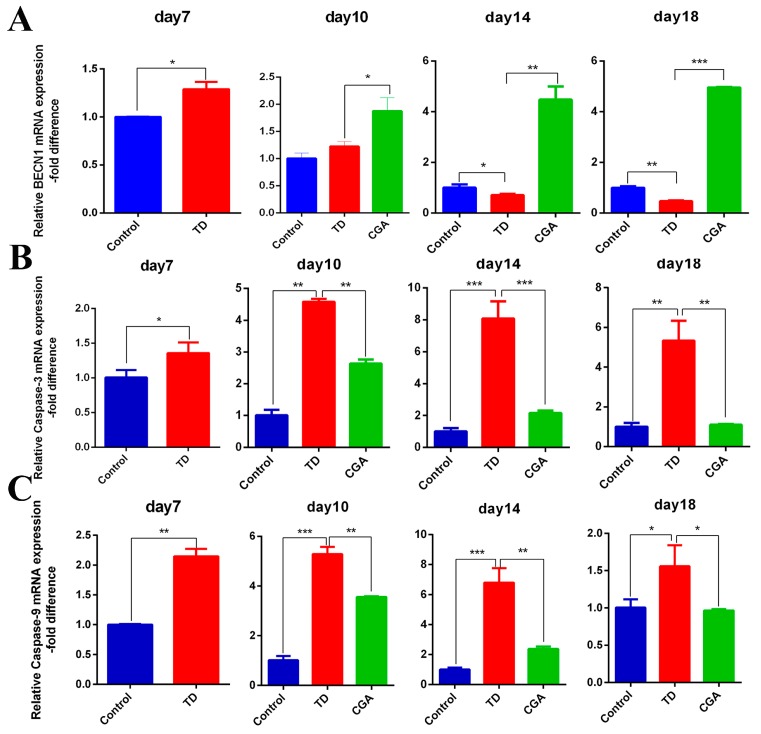
CGA regulates expression of apoptotic and autophagic genes in growth plate. (**A–C**) The relative mRNA expression of *BECN*1, Caspase-3, caspase-9 in tibial growth plate were measured by RT-qPCR at days 7, 10, 14 and 18. GAPDH was used as an internal control. All of the data represent means ± SD of three independent experiments. * *p* < 0.05, ** *p* < 0.01, *** *p* < 0.001.

Gene expression of caspase-3, caspase-9, *BECN*-1 were confirmed by RT-qPCR. Our results showed that mRNA expression of pro-apoptotic genes caspase-3 and caspase-9 are significantly increased in TD group compared with the control group at days 7, 10, 14, and 18. The mRNA expression of autophagic gene *BECN*1 in the TD group were significantly lower than in the TD group from day 14. In contrast, after application of chlorogenic acid from day 7, the expression of caspase-3 and caspase-9 began to decline and it significantly decreased from day 10 (especially on day 14) compared with the TD group. The expression level of *BECN*1 in the CGA group had a continuous raised tendency and a significant difference appeared at day 10 compared with the TD group. Obviously, the expression of apoptotic genes caspase-3 and caspases-9 gradually approached the expression level of the control group until day 18 (Figure 6).

### 2.7. CGA Regulates Expression of ECM Catabolic Genes in Growth Plate

**Figure 7:**
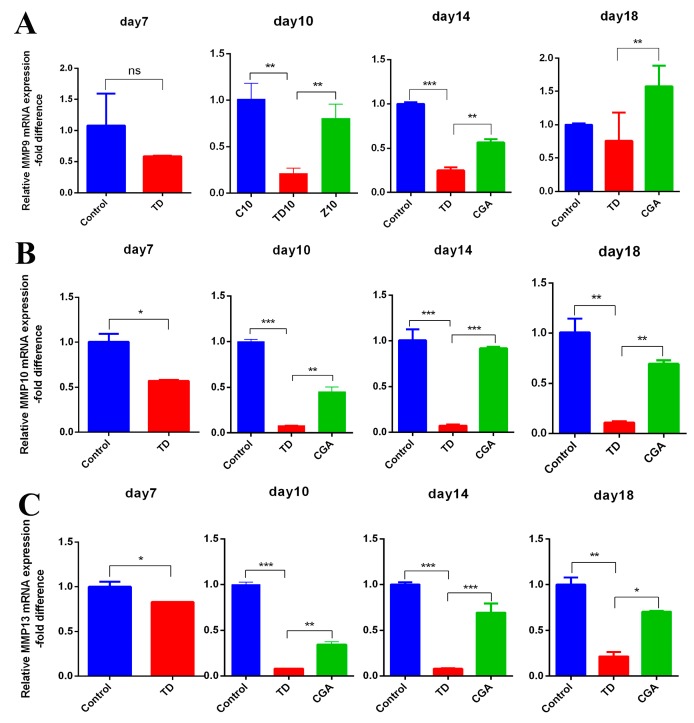
CGA regulates expression of ECM catabolic genes in growth plate. (**A**–**C**) The relative mRNA expression of *MMP*9, *MMP*10, *MMP*13 in tibial growth plate were measured by RT-qPCR at days 7, 10, 14 and 18. GAPDH was used as an internal control. All of the data represent means ± SD of three independent experiments. * *p* < 0.05, ** *p* < 0.01, *** *p* < 0.001.

To investigate the effect of CGA on ECM catabolism, we chose some representative genes from matrix metalloproteinase, *MMP*-9, *MMP*-10, and *MMP*-13. The expression of *MMP*s we chose significantly down-regulated in TD group from days 10 to 14 when compared with the control group, *MMP*-10 and *MMP*-13 showed more obvious reduction. The administration of CGA resulted in a significant up-regulation of these three *MMP*s genetic expression in the CGA group as compared with the TD group. The expression of *MMP*-10 and *MMP*-13 in CGA group became similar to the control group, but the expression of *MMP*-9 is still high (Figure 7).

### 2.8. CGA Regulates Protein Expression of BECN1, CASP9 and MMP13 in Growth Plate

**Figure 8:**
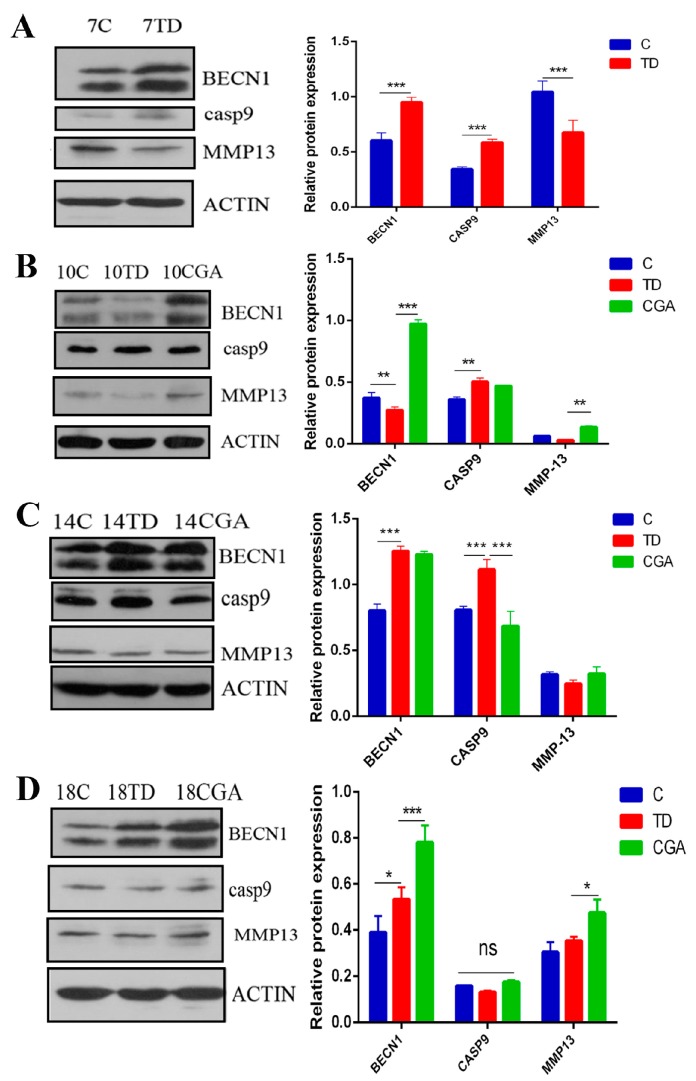
CGA regulates protein expression of BECN1, CASP9 and MMP13 in growth plate. (**A**–**D**) The relative protein expression of BECN1, CASP9, MMP13 in tibial growth plate were measured by western blotting at days 7, 10, 14 and 18. ACTIN was used as an internal control. All of the data represent means ± SD of three independent experiments. * *p* < 0.05, ** *p* < 0.01, *** *p* < 0.001.

The protein expression of BECN1, CASP9 and MMP13 were assessed by western blotting on days 7, 10, 14 and 18. The expression level of the pro-apoptotic protein, CASP-9 was significantly enhanced in the TD group, whereas the expression of MMP13 protein was reduced on days 7, 10, 14, 18. However, the administration of CGA significantly decreased the expression level of CASP-9 on day 14 and obviously increased the level of MMP13 on day 10 compared with the TD group. The expression of BECN1 was down-regulated in the TD group on day 10, whereas CGA up-regulated the level of BECN1 significantly from day 10 to day 18 compared with the control group (Figure 8).

## 3. Discussion

In recent years, Chinese traditional medicines have come to the attention of scientists due to their potential for widespread application, fewer side effects, and easy availability. Various chemical components from Chinese traditional medicines have been reported to have potential to suppress cancer and ameliorate rheumatoid arthritis [22]. Chlorogenic acid is the major component that is extracted from the leaf of Eucommia. Studies in recent years have also suggested that CGA possesses protective effects on osteoporosis in ovariectomized rats through the Shp2/PI3K/Akt pathway.

In the current study, we first used chlorogenic acid to treat tibial dyschondroplasia, during the animal experiment, we observed reduced body development because of TD as previous research depicted [23], but chickens with TD gradually recovered and became vigorous in CGA treatment group. The body weight of broilers in the CGA treatment group began to exceed the TD group from day 14. In addition, the ADFI and ADWG of chickens in the TD group also decreased, whereas these two parameters had been improved by CGA. But interestingly the FCR of chickens in TD group was higher than control group and CGA group, in consideration of the low body weight and low feed intake, this may indicate that the resorption of nutrients in TD afflicted chickens is compromised. We also measured specific parameters of tibial development, as expected, CGA reversed the situation of short tibial length and low weight of tibia because of TD. All our findings demonstrated the positive effect of CGA on broiler growth and the facilitation effect on skeleton growth in vivo.

During the development of growth plate, the terminally differentiated chondrocytes must be eliminated to guarantee the thickness of normal growth plate. They seem to produce additional proteinases like MMP-13 and undergo apoptosis. At the same time, the inner perichondrium cells are thought to transfer to osteoblasts [24]. However, in the maturation region of metaphysics, dead cells rarely exist, which indicates that osteoblasts can finish their whole life history, perhaps from osteoblasts to osteocytes [25]. Although many reports have shown that apoptosis plays an important role in development, many studies still suggest that apoptosis participates in the pathogenesis of diseases [26], for example, the dysregulation of chondrocyte apoptosis played a critical role in the pathogenesis of osteoarthritis [27]. The fact that apoptosis existed in TD-affected growth plate was described in previous reports, and TUNEL-stained chondrocytes were observed around blood vessels [25]. However, the mechanisms of apoptosis which may be responsible for TD still remain unclear. Caspases are a family of enzymes playing unignorable roles in apoptosis, from which capsase-3 is an executioner and caspases-9 is an initiator. It has been illustrated that pro-apoptotic caspases are important in odontogenesis [28]. As the result of our experiment, the expressions of caspases-3 and caspases-9 were significantly raised in the TD group from days 7 to 18, which indicated the enhancement of apoptosis, then the apoptotic cells accumulated. That is in general agreement with previous report in which apoptosis chondrocytes were observed in distal area of growth plate affected by TD, especially in severe lesions [29]. In some surveys, it was also considered that the absent accessibility of phagocytic cells (responsible for the removal of apoptotic cells) to penetrate avascular cartilage can lead to the development of cartilage plug in growth plate [29]. Our study demonstrated that the expression level of caspases was restored by the administration of chlorogenic acid from day 10 as compared with the TD group.

*BECN*1 is an important gene playing a critical role in autophagy, which has a complex relation with apoptosis. In our experiment, we tested the expression of BECN1 in different days among the control, TD and CGA groups. As a result, the expression of BECN1 has an adverse variation tendency with the development of TD, and the administration of CGA significantly increased the expression level of BECN1 compared with TD groups. This expression variation of BECN1 and the improvement of tibial index collected from TD affected chickens in CGA groups are consistent with previous report, in which researchers point that BECN1 is important in skeletal development and benefits the mineralization of bone [30]. Moreover, BECN1 has a relation with MMPs in many diseases, such as endometriosis [31] and tongue squamous cell carcinoma [32], but the relation between them in TD still remains vague. There are several lines of evidence which show that MMPs occupy an important part in endochondral ossification through promoting angiogenesis and remodeling extracellular matrix. The MMPs we chose in our experiment are MMP-9, gelatinase B; MMP-10, stormelysin; and MMP-13, collagenase. VU TH suggests that MMP-9 plays an important role in angiogenesis of growth plate [33], besides, MMP-9 also has been proven as one of the earliest proteinases which is involved in the recovery of TD [34]. MMP-13, collagenase-3, can catalyze collagen I and II, and it has a connection with other matrix metalloproteinases. The mutations of human MMP-13 can cause recessive metaphysical dysphasia [35]. In the current research, the expression of MMP-9, MMP-10, MMP-13 was significantly decreased in TD groups compared with control groups, especially on days 10 and 14. This finding is consistent with previous reports [36] that CGA significantly increased their expression, at the same time, the levels of BECN1 had also been improved. Moreover, we can find that there is a similiar increasing tendency between the expression of MMPs we chose and BECN1 on different days in CGA groups. 

MMP has been reported as an important enzyme involved in normal endochondral ossification, and also affects the development and recovery of TD. MMPs play an important role in the degradation of extracellular matrix. In our study, we find that lots of matrices failed to degrade in the TD group. These enzymes can be secreted by epithelial cells, endothelial cells, vascular endothelial cells, chondrocytes and macrophages [37]. The apoptotic cells accumulated in growth plate can lead to the decrement of MMPs due to the absence of phagocytes. Moreover, the ECM cannot be cleaved and the biologically activated molecules such as VEGFA can’t be released because of the decrease of MMPs (especially MMP-9 and MMP-13) [38]. This phenomenon can finally aggravate the apoptosis of growth plate and lead to the formation of avascular cartilage plug. Another type of programmed cell death, autophagy, occurred in growth plate can benefit cell survival [39]. There are many reports that demonstrate that BECN1 can be a novel substrate of caspases. Caspases can directly participate in apoptosis, and after the cleavage of BECN1 by caspases, the production of BECN1 fragments can lose the ability to induce autophagy and get the potential to induce apoptosis [40]. Thus, from our results we speculate that because of the unregulated caspases, apoptotic cells accumulated, and the cleavage of BECN1 can aggravate the development of apoptosis, then secretion of MMPs became down-regulated because of the death of vascular endothelial cells and absence of phagocytes. As a result, the cleavage and remodeling of ECM had been delayed and the calcification of growth plate suspended. CGA which we applied in this experiment may recovered TD via reversing the process we inferred above. Of course, the mechanisms of TD still need to be proven by additional studies, but our experiment offers new insights to help other scientists figure out the mechanisms of TD. Further, our study can also enlarge the application scope of CGA and increase the knowledge of scientists about Chinese traditional medicines.

## 4. Materials and Methods 

### 4.1. Chickens Husbandry and Animal Ethics

A group of 150 one-day-old healthy Arbor Acres chickens (AACs) were purchased from a commercial hatchery (Chia Tai Animal Husbandry Co. Ltd., Wuhan, China). The chickens were raised in metal cages for 18 days and provided with the recommended breeding temperature and clean environment. Adequate feed and water were provided ad libitum.

Animal experiments were approved by the Ethical Committee of the Huazhong Agricultural University (Permit No. 4200695757, 6 March 2017) and accorded with the guidelines from the Laboratory Animal Research Center, Wuhan, China.

### 4.2. Animal Experiment and Production Indicator Analysis

**Figure 9:**
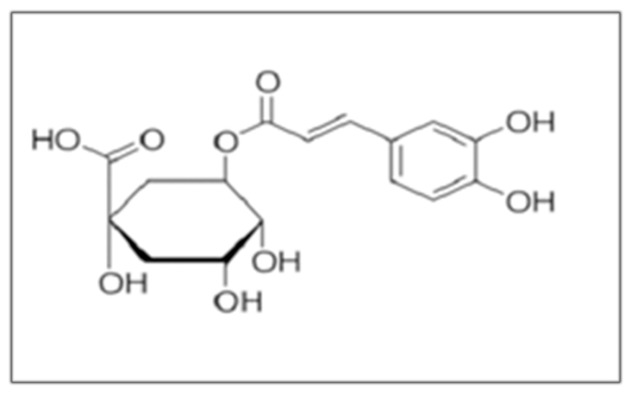
Chemical structure of chlorogenic acid.

The chemical structure of CGA was shown in Figure 9. All the broilers were divided randomly into three groups with 50 chickens in each group: control group, TD group and CGA group. The control group was offered normal diet and the experimental groups (TD and CGA groups) were fed the same diet as control but with the addition of 100 mg/kg thiram (which was added from days 4 to 7). After the induction of TD on day 8, CGA group began to apply 45 mg/kg/day chlorogenic acid solution to chickens until day 18 [21]. 

During the animal experiment, the chickens in one cage as a group were weighed, the results divided by the number of chickens were recorded as BW (body weight). ADFI (Average daily feed intake) and ADWG (average daily weight gain) were also calculated and recorded. In the end, the feed conversion ratio (FCR) was calculated.

### 4.3. Sample Collection and Evaluation of Tibial Parameters

Twelve chickens from every group were randomly selected to be slaughtered on days 7, 10, 14 and 18. All chickens were euthanized after injecting pentobarbital (25 mg/kg). Before the sacrifice, blood samples were collected for biochemical analysis. After slaughtering, liver and tibial specimens were separated and tibial parameters including tibia weight, tibia length, width of tibia growth plate were measured by an electronic balance sensitive to 0.001 g and a digital caliper purchased from TATA Company (Shanghai, China), respectively. Then, some of tibia bones were fixed in paraformaldehyde (4%) and then soaked in tubes filled with decalcifying fluid (10%) for further H&E (Hematoxylin and Eosin) staining etc. The other tibiae were frozen instantly in liquid nitrogen and stored at –80 °C fridge for further analysis.

### 4.4. Biochemical Analysis

The blood samples were centrifuged at 3500× g for 15 min in order to separate the serum. Then, the serum was stored at –20 °C. The concentration of malonic dialdehyde (MDA) and vitality of total superoxide dismutase (T-SOD) and total antioxidant capacity (T-AOC) in serum samples were measured by commercial assay kits purchased from the Institute of Biological Engineering Inc., Nanjing, China via ultraviolet spectrophotometer according to the instruction of manufactures.

### 4.5. Hematoxylin and Eosin (H&E) Staining

Samples were fixed overnight in 4% paraformaldehyde or 2.5% glutaraldehyde in phosphate-buffered saline (PBS) at 4 °C. Serial histological sections (4-µm thickness) were prepared after samples had been fully decalcified in 10% ethylenediaminetetraacetic acid (EDTA) decalcifying fluid, dehydrated, embedded in paraffin wax, and then stained with hematoxylin and eosin. Sections were examined by light microscopy [41].

### 4.6. RNA Extraction

The total RNA of every growth plate detached from individual chickens was extracted using a Trizol reagent (Life Technologies, Carlsbad, CA, USA) according to an efficient RNA extraction method. The RNA integrity was verified by denaturing formaldehyde gel electrophoresis and the concentration was measured by Nanodrop 2000 analyzer (Thermo Fisher Scientific, Shanghai, China).

### 4.7. qRT-PCR

After extraction of total RNA, a EasyScript One-Step gDNA Removal and cDNA Synthesis Kit purchased from TransBionovo Co., Ltd. (Beijing, China) was used to synthesize cDNA according to the instructions of the manufacturer. Then qRT-PCR was performed using TransStart Tip Green qPCR Kit in a Step One-Plus™ Real-Time PCR System purchased from Applied Biosystems (Foster City, CA, USA). All reactions were repeated three times. The relative mRNA expression of each gene was normalized against the expression of gene GAPDH which was used as an internal control by the ∆∆^CT^ calculation method. The primers used in this process are shown in Table 1.

### 4.8. Western Blotting Analysis

Tibia growth plates were homogenized in ice-cold buffer and incubated at 4 °C for two hours. The samples were centrifuged at 12,000 rpm for 10 min in order to collect the supernatant (total protein) and the concentration was determined using a BCA protein quantitative detection kit (Servicebio technology, Wuhan, China). Equal proteins from different samples were separated by SDS-PAGE via 12% polyacrylamide gel until the dye band reached the end of the gel, after that, the gel was transferred to PVDF membranes, which were incubated in 5% skimmed milk for two hours. The membranes were incubated overnight at 4 °C with rabbit monoclonal anti-BECN1, anti-CASP-3, anti-CASP-9 and anti-MMP-13 primary antibodies (ABclonal technology, Wuhan, China) and then were incubated with secondary antibody (1:3000 dilution) (HRP labeled rabbit anti-goat secondary antibodies) for 1 h at room temperature. After washing, the bands were visualized and exposed by chemiluminescence and radiography film, respectively. The images were taken using an imaging system (EPSON, China, #V300).

### 4.9. Statistical Analysis

All of pictures were finished by using Graphpad Prism 6, and the results were evaluated using the two-way ANOVA and Student’s t-test via SPSS (Version 17.0. Chicago, IL, USA). Significant differences between the two groups were considered to have *p* values < 0.05 and the data in the figures are presented as means ± SD.

## Figures and Tables

**Table 1:** Primers uesd for qRT-PCR.

Name	Primer Sequence (5′-3′)	Accession Number	Product Size
Beclin1	CGACTGGAGCAGGAAGAAG	NM_001006332.1	115 bp
	TCTGAGCATAACGCATCTGG		
MMP-9	ATGAACTACTCCCCCGACCTG	NM_204667.1	258 bp
	AGTCCAGAACTCATCATCATCG		
MMP-10	TGCTTCTGGATTTCACGGTG	NM_001278089.1	101 bp
	AGTGGGCATCCCCTCCTATC		
MMP-13	CAACCCAAAACATCCCAAAAC	NM_001293090.1	258 bp
	CCATTCATAGCCCAAACCTTC		
Caspase-3	ACTCTGGAATTCTGCCTGATGACA	NM_204725.1	129 bp
	CATCTGCATCCGTGCCTGA		
Caspase-9	ATTCCTTTCCAGGCTCCATC	XM_424580.6	130 bp
	CACTCACCTTGTCCCTCCAG		
GAPDH	CCTTCATTGACCTTCACTACATGGTCTA	NM_204305.1	127 bp
	TGGAAGATGGTGATGGCCTTTCCATTG		

## Abbreviations

CGA	Chlorogenic acid
TD	Tibial dyschondroplasia
ECM	Extracellular Matrix
